# Poly-Gamma-Glutamic Acid (γ-PGA)-Based Encapsulation of Adenovirus to Evade Neutralizing Antibodies

**DOI:** 10.3390/molecules23102565

**Published:** 2018-10-08

**Authors:** Ibrahim R. Khalil, Martin P. Khechara, Sathishkumar Kurusamy, Angel L. Armesilla, Abhishek Gupta, Barbara Mendrek, Tamara Khalaf, Mariastella Scandola, Maria Letizia Focarete, Marek Kowalczuk, Iza Radecka

**Affiliations:** 1 Wolverhampton School of Sciences, Faculty of Science and Engineering, University of Wolverhampton, Wulfruna Street, Wolverhampton WV1 1LY, UK; Ibrahim.khalil@wlv.ac.uk (I.R.K.); mpkhechara@wlv.ac.uk (M.P.K.); S.K.Kurusamy@wlv.ac.uk (S.K.); A.Armesilla@wlv.ac.uk (A.L.A.); a.gupta@wlv.ac.uk (A.G.); khalaft@wolvcoll.ac.uk (T.K.); 2 Department of Biology, College of Science, Tikrit University, Tikrit PO Box 42, Iraq; 3 Centre of Polymer and Carbon Materials, Polish Academy of Sciences, ul. M. Curie-Skłodowskiej 34, 41-819 Zabrze, Poland; bmendrek@cmpw-pan.edu.pl; 4 Department of Chemistry ′G. Ciamician′ and National Consortium of Materials Science and Technology (INSTM, Bologna RU), Alma Mater Studiorum–Università di Bologna Via Selmi 2, 40126 Bologna, Italy; mariastella.scandola@unibo.it (M.S.); marialetizia.focarete@unibo.it (M.L.F.)

**Keywords:** biodegradable polymer, γ-PGA, chitosan, adenovirus, immunogenicity

## Abstract

In recent years, there has been an increasing interest in oncolytic adenoviral vectors as an alternative anticancer therapy. The induction of an immune response can be considered as a major limitation of this kind of application. Significant research efforts have been focused on the development of biodegradable polymer poly-gamma-glutamic acid (γ-PGA)-based nanoparticles used as a vector for effective and safe anticancer therapy, owing to their controlled and sustained-release properties, low toxicity, as well as biocompatibility with tissue and cells. This study aimed to introduce a specific destructive and antibody blind polymer-coated viral vector into cancer cells using γ-PGA and chitosan (CH). Adenovirus was successfully encapsulated into the biopolymer particles with an encapsulation efficiency of 92% and particle size of 485 nm using the ionic gelation method. Therapeutic agents or nanoparticles (NPs) that carry therapeutics can be directed specifically to cancerous cells by decorating their surfaces using targeting ligands. Moreover, in vitro neutralizing antibody response against viral capsid proteins can be somewhat reduced by encapsulating adenovirus into γ-PGA-CH NPs, as only 3.1% of the encapsulated adenovirus was detected by anti-adenovirus antibodies in the presented work compared to naked adenoviruses. The results obtained and the unique characteristics of the polymer established in this research could provide a reference for the coating and controlled release of viral vectors used in anticancer therapy.

## 1. Introduction

Cancer is still the second leading cause of death in developing and developed countries [1,2]. Despite the increasing rate of survival in the last 40 years, the severe side effects of radiation and chemotherapy cancer treatment have been acknowledged to be of major importance, and could cause a number of problems, including systemic toxicity, mild cognitive impairments, and mouth ulcerations [3]. Moreover, long-term childhood-cancer survivors’ follow-up studies have observed the incidence of many side effects, such as endocrine disruption, cardiac function, auditory dysfunction, and the possibility of developing other types of cancer due to long-term chemotherapy treatment [4], let alone the recurrence of the disease.

However, new types of treatment have been established. These new approaches include gene therapy [3,5], nanoparticulate vectors [6,7,8], modified bacteria [9,10], and the utility of the immune system [11,12]. Recently, several approaches have been conducted using no-viral anticancer therapy. Cheng et al. (2018) used a self-assembled supramolecular host–guest delivery system to deliver chemotherapeutics to drug-resistant cancer cells and tumors [13]. In another study by Liu and coworkers (2018), a supramolecular hydrogel was used for drug-resistant cancer therapy [14]. Moreover, viruses that have the ability to replicate specifically inside cancer cells and subsequently causing death to these infected cells have shown great promise; these are called oncolytic viruses [15,16,17,18]. In recent years, there has been an increasing interest in oncolytic adenoviral vector anticancer therapy due to these viruses’ ability to efficiently infect a variety of both dividing and nondividing cells, and because of their high-affinity binding site for attaching to the coxsackie virus and adenovirus (Ad) receptor (CAR) of susceptible cells. Ad can also easily produce high titers in vitro. By simply replacing part of the genome of the virus with a therapeutic gene, viruses (retrovirus and adenovirus) can also be used as gene-delivery carriers. They have been widely used in reported gene-delivery studies and in the majority of ongoing clinical trials due to their efficiency in delivering genetic materials [19]. However, the induction of an immune response is the major barrier towards Ad vector applications. This limitation results in the reduction of viral transduction and thus decreased therapeutic efficiency [20,21].

Several attempts have been made to overcome the induction of immune responses, such as the use of pharmacological inhibitors, genetic engineering, and carrier cells. All these strategies significantly decreased overall inflammatory response, yet did not inhibit the production of neutralizing antibodies [22]. More recently, the use of biopolymers as drug-delivery systems for anticancer therapy have received wide attention because of their nonimmunogenicity, nontoxicity, and biodegradability. One such biopolymer is poly-gamma-glutamic acid (γ-PGA). Polymer γ-PGA is a biocompatible, biodegradable, nontoxic, and nonimmunogenic biopolymer [23,24,25,26]; **γ**-PGA is a homopolyamide composed of glutamic acid monomers connected by amide linkages between α-amino and **γ**-carboxyl groups, with D and L isomeric units distributed in repeated blocks [27,28]. The γ-PGA biopolymer has been widely used as a drug-delivery platform because of its free carboxyl groups on the side chains in the α-position, which offers attachment points for the conjugation of therapeutic agents. This enhances aqueous solubility and controls the release of the drug [23,29].

One long-term goal is to develop ‘stealth’ vectors of low immunogenicity carrying destructive cargoes capable of destroying the replication abilities of, for example, the Epstein–Barr virus (EBV), the major cause of Hodgkin′s lymphoma [30]. The main aim of this study was to see if polymer encapsulation of such vectors helps to lower their immunogenicity.

## 2. Results

### 2.1. γ-PGA Identification and Characterization

**Figure 1:**
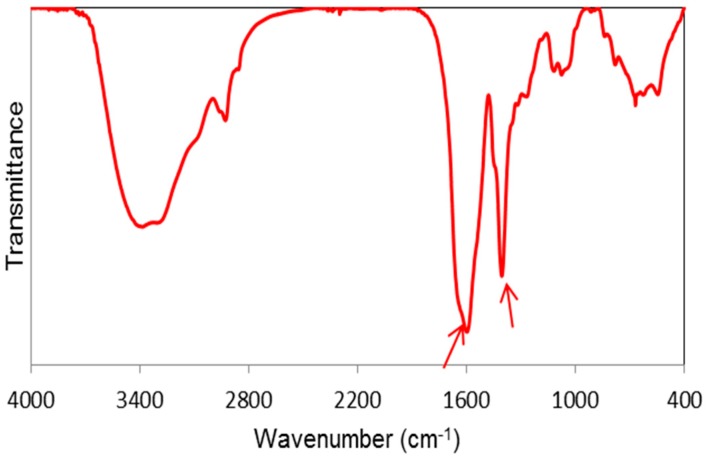
Fourier transform infrared (FT-IR) spectra of poly-gamma-glutamic acid (γ-PGA). The arrows indicate typical absorptions of carboxylic group.

Different analytical tools were applied to identify and characterize γ-PGA produced by *Bacillus licheniformis* via a fermentation route. The produced γ-PGA was water-soluble. High-performance liquid chromatography (HPLC) results revealed that the L-monomer content in the γ-PGA hydrolyzate was 15%, while the content of d-monomer was as high as 85%. The number average molar mass (M*_n_*) of produced biopolymer analyzed by gel permeation chromatography (GPC) was 626,000 Da, and dispersity was 1.7. The Fourier transform infrared (FT-IR) spectrum of γ-PGA is shown in Figure 1, where the bands of the carboxylic group, in its acidic COOH or ionized COO-form, are marked by arrows. The results indicate that γ-PGA is mostly in salt form.

**Figure 2:**
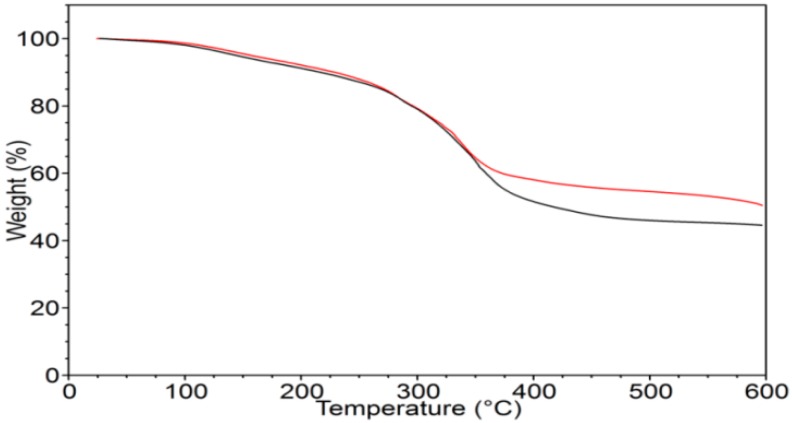
Thermogravimetric (TGA) curves of γ-PGA in nitrogen (black curve) and in air (red curve).

Figure 2 shows the TGA curves of γ-PGA in an inert environment (N_2_) and in air. The two curves are quite similar and show continuous weight loss; the initial one (starting at around 100 °C) is likely to be associated with the evaporation of absorbed water. The overall thermogravimetric (TGA) behavior indicates that the sample is thermally unstable in both environments, with a residue at 600 °C, higher than 40%.

**Figure 3:**
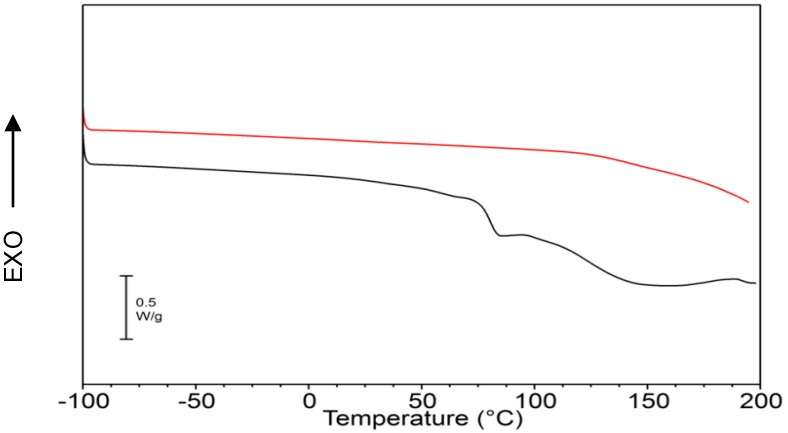
Differential scanning calorimetry (DSC) curves of γ-PGA: first heating scan (black curve) and second scan after quench-cooling from the melt (red curve).

The result from X-ray diffraction (XRD) analysis (result not shown) proved that γ-PGA produced by *B. licheniformis* was amorphous. This result is confirmed by the calorimetric analysis shown in Figure 3.

The first DSC heating scan of γ-PGA (black curve) shows a baseline shift attributed to the glass transition around 80 °C that is followed by a broad endotherm associated with water evaporation. After quench-cooling, a second heating scan was performed (see DSC curve in Figure 3) and in such scan no endotherm due to water evaporation was evident any more. γ-PGA exhibits a continuous baseline drift likely due to thermal degradation. The T_g_ of γ-PGA, expected to be higher than that in the first scan due to the fact that water acts as a plasticizer, was not detectable in the second scan.

**Figure 4:**
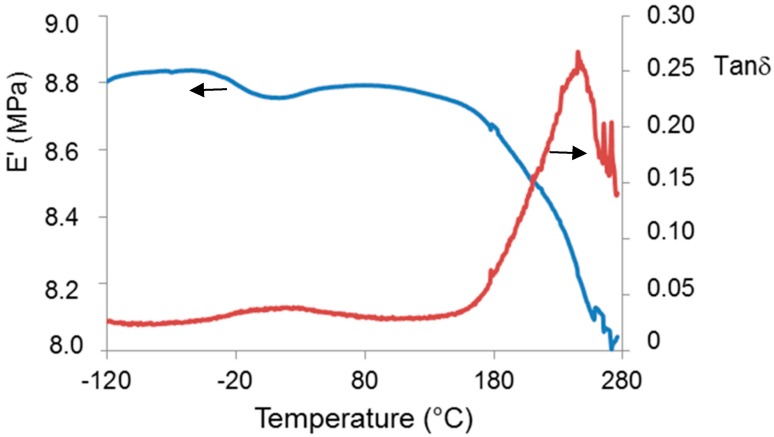
Dynamic–mechanical curves (red curve: loss tangent, tan δ; blue curve: storage modulus, E’) of γ-PGA.

The dynamic mechanical spectrum of γ-PGA isolated from by *B. licheniformis* is shown in Figure 4, where storage modulus (E’) and loss tangent tan δ were reported as a function of temperature.

**Figure 5:**
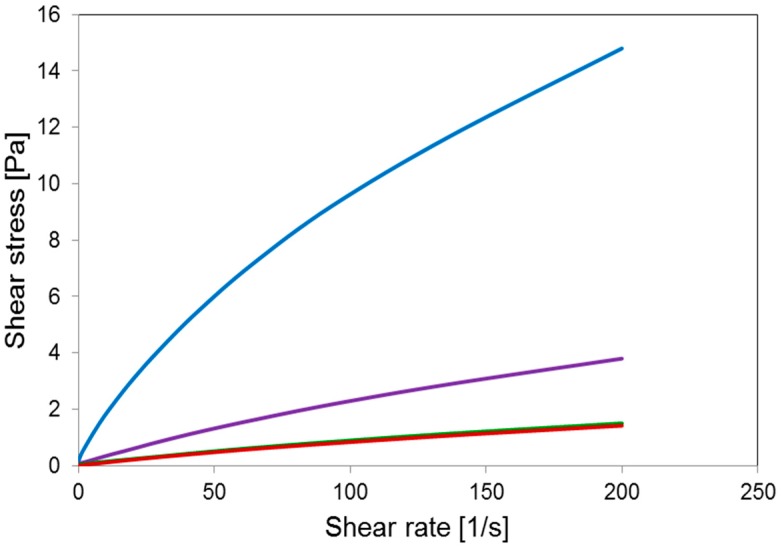
Shear stress vs. shear rate curves of water solutions of γ-PGA with different concentrations: 2% (blue), 0.5% (violet), 0.25% (green), and 0.125% (red).

A main relaxation process was observed, displayed as a steep drop of the storage modulus and a peak of loss tangent, associated with the glass transition that starts above 100 °C and is most probably affected by concomitant thermal degradation processes (highlighted by TGA, Figure 2). A secondary local-mode relaxation centred around room temperature was also observed. Above the latter relaxation, the modulus was seen to increase, indicating an increase of sample rigidity due to the evaporation of the absorbed water. The rheological behavior of the water solutions of γ-PGA at different concentrations is shown in Figure 5.

At low shear rate, in the linear viscoelasticity range, the behavior of polymer solutions is expected to be linear, implying constant viscosity. For the analysed system, this was true only at low concentrations. Behavior at high concentration (c ≥ 0.5%) suggests the presence of macromolecular aggregation at low rate regimes. Such interactions tend to loosen with increasing shear rate.

**Figure 6:**
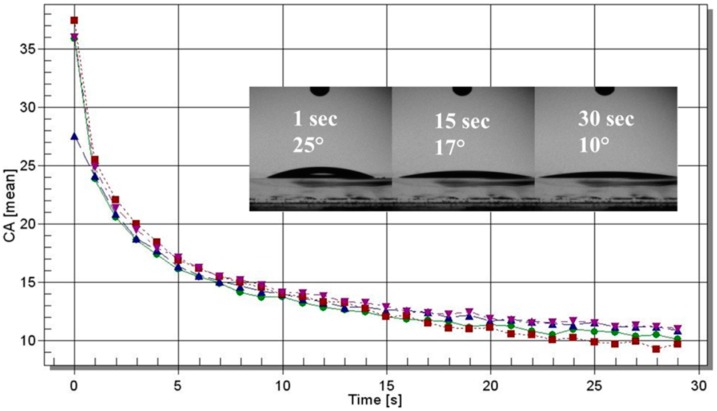
Contact angle of water on the surface of γ-PGA as a function of time. Four sets of data were plotted (the four curves with different colours refer to four drop tests). Pictures of the drop after 1, 15, and 30 s are shown in the insert.

Polymer γ-PGA was coated on a glass slide via spin coating and the results of water-contact angle measurements are shown in Figure 6. The values of contact angle (θ) are always well below 90°, showing the hydrophilic nature of the surface. After drop deposition on the polymer surface, θ decreased with time due to strong polymer–water interaction. The average contact angle after 30 s was θ = 10.4° ± 0.5°.

There are fundamental reasons for using γ-PGA produced by *B. licheniformis* 9455a as a viral vector. First, the higher the content of d-glutamic acid monomer is, the less the immunogenic polymer, and it was demonstrated that *B. licheniformis* 9945a produces a capsule of γ-PGA composed of 85% of d-glutamic acid monomer. This capsule produced by *B. licheniformis* 9945a has already been used as a surrogate for *B. anthracis* capsules in many studies [31,32], which are composed of d-γ-PGA and known to be anti-phagocytic. Second, γ-PGA polymer produced by this strain is amorphous, and amorphous polymers are more hydrophilic, which makes them much more preferable in drug-delivery systems than the crystalline forms, as the latter are not generally accessible [33].

### 2.2. Nanoparticle Characterization

**Figure 7:**
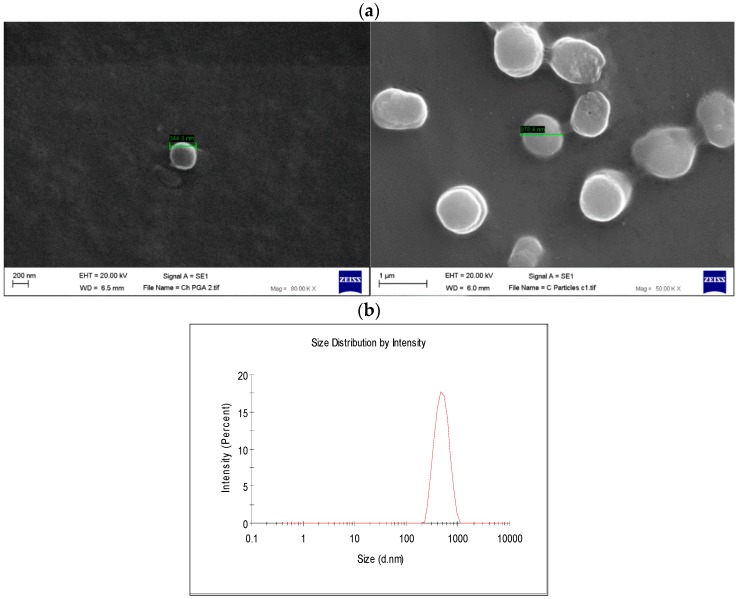
(**a**) Scanning electron microscopy (SEM) images of γ-PGA nanoparticles (NPs); (**b**) Dynamic light-scattering (DLS) analysis of γ-PGA NP formed by ionic gelation method; γ-PGA NPs formed by the formulation of (0.05:0.15 CH:γ-PGA *w*/*v*). Magnification power is 80,000× (left image) and 50,000× (right image).

Stable and self-assembled polyelectrolyte nanoparticles (NPs) were formulated using a gelation method by the ionic interaction between γ-PGA carboxylic groups and linear chains of chitosan (0.05:0.15 CH:γ-PGA ratio). Chitosan is known to enhance NP hydrophilic properties and control their surface charge. The sizes and the zeta potential values of NPs were measured by dynamic light scattering (DLS) using a Zetasizer. The mean size of γ-PGA-CH NPs was 485.8 ± 2.3 nm and the net surface charge of the particles was negative (−23.7 ± 1.4 mV). Surface morphology and particle size were further investigated using scanning electron microscopy (SEM) (Figure 7a). SEM images revealed that the γ-PGA-CH NPs were monodispersed and almost spherical in shape, with a smooth surface and submicron size, which is in accordance with the particle size observed by DLS (Figure 7b). No aggregation was observed at the tested concentration. Particles suspended in deionised water showed visual stability with no aggregation or precipitation even after 14 days at 4 °C.

### 2.3. Adenovirus Encapsulation and Release Profile

**Figure 8:**
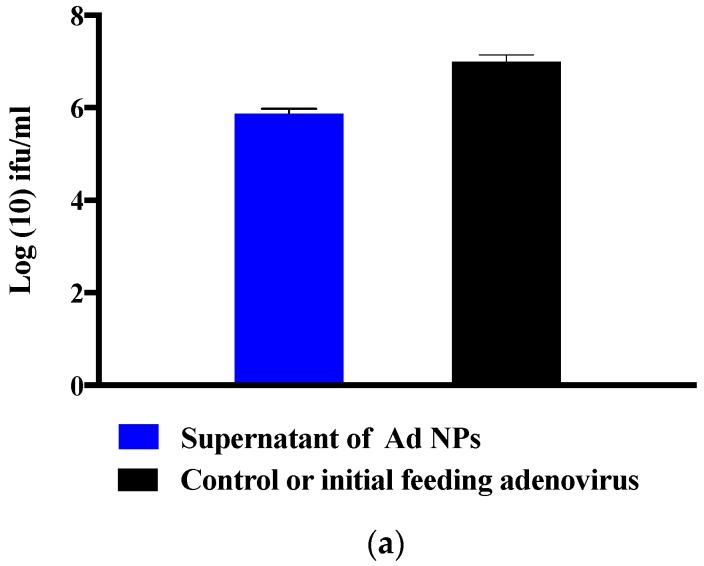

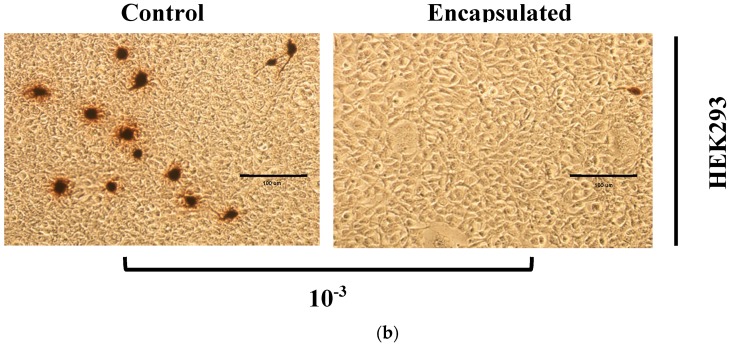
Encapsulation efficiency (EE) of adenovirus into γ-PGA-chitosan NPs; (**a**) EE evaluated by measuring the difference of Ad infectivity in the supernatant and the control. Difference refers to the amount of encapsulated Ad inside the particle, which means the pellet after centrifugation containing Ad. (**b**) HEK293A cells infected with supernatant containing Ad from the same dilution of the control and NP suspension. Black dots represent cells infected with Ad, and titers were calculated according to the Adeno-X Rapid Titter Kit manufacturer instructions. Magnification power ×200, scale bar 100 µm. Experiments were conducted in triplicate (*n* = 3).

Ad encapsulation efficiency (EE) was around 93% (Figure 8a) after converting the logarithmic values to percentages. This was confirmed when cells were infected with supernatant containing Ad from the same dilution of the control and NP suspension. Figure 8b represents cells infected with Ad, and titers were calculated according to the Adeno-X Rapid Titer Kit manufacturer instructions.

**Figure 9:**
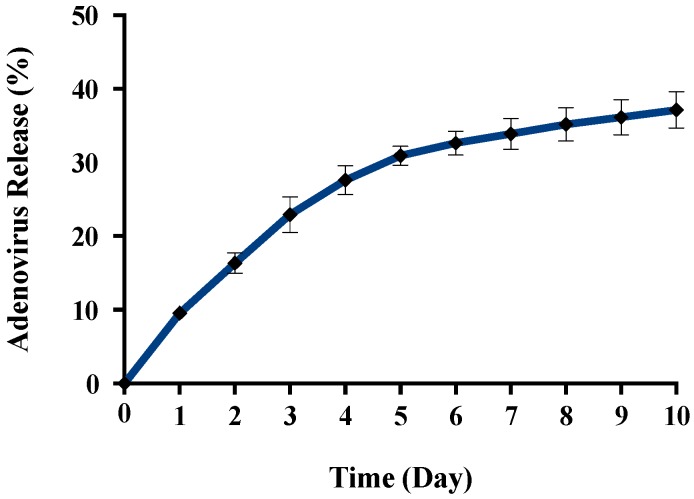
In vitro release of encapsulated adenovirus into γ-PGA-chitosan NPs. The release was performed in a DMEM medium at 37 °C in orbital shaker at 150 rpm for 10 days. Experiments were conducted in triplicate (*n* = 3).

The purpose of this investigation was to gain a better understanding about the release mechanism. Release of the encapsulated Ad phage was performed in a Dulbecco’s Modified Eagle’s Medium (DMEM) medium (pH = 7.0) at a concentration of 5 mg/mL. It was noticed that there was a burst release of Ad (9.5%) from γ-PGA-CH NPs at day 1, followed by an additional release of 16.3% on the second day. It was observed that the release was sustained until day 5 (Figure 9). Subsequently, the release slowed to a maximum value of 37.3%. The experiment was stopped after 10 days due to aggregation of NPs during centrifugation/resuspension cycles.

### 2.4. Adenovirus Immunogenicity 

**Figure 10:**
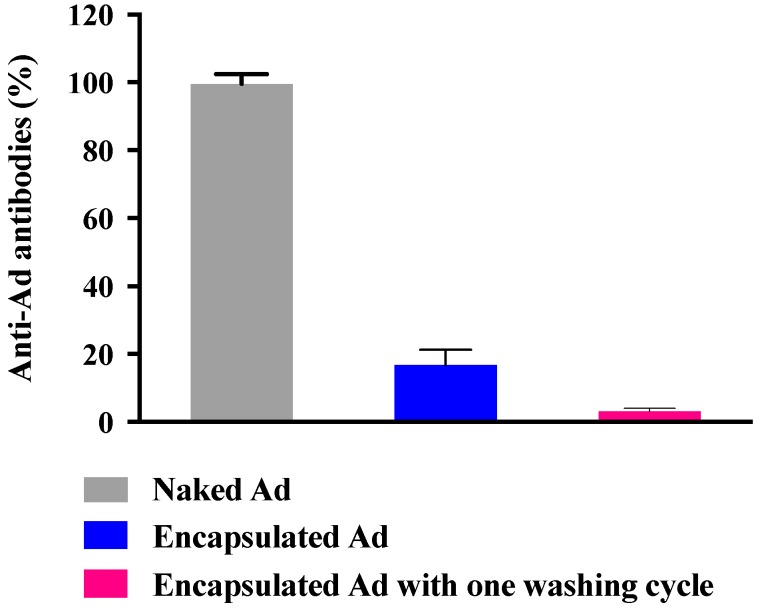
Hexon ad-specific ELISA antibodies. Antibodies were inoculated in vitro with encapsulated Ad and naked Ad. Experiments were conducted in triplicate (*n* = 3).

In order to determine whether NPs have the potential to shield Ad from neutralizing antibodies or not, the encapsulated Ad in the γ-PGA-CH NPs were exposed to antihexon antibodies. The detection of antibodies was evaluated and compared to the recognition of naked Ad. This investigation was performed in 96-well plates, seeded with the same titration of encapsulated and naked Ad (9.8 × 10^6^ ifu/mL), and measured using the ELISA technique. The absorbance of the resulting solution was measured at 450 nm. Results indicated that antiadenovirus hexon-protein antibodies were able to recognise all naked Ads when presented to them. In contrast, the antiadenovirus hexon-protein antibodies were only able to recognise 16% of the encapsulated Ad in γ-PGA NPs. However, when the particles were subjected to a centrifugation/washing cycle, the amount of hexon protein detected was reduced to 3.1% (Figure 10).

### 2.5. Fluorescent Labeled NPs

**Figure 11:**
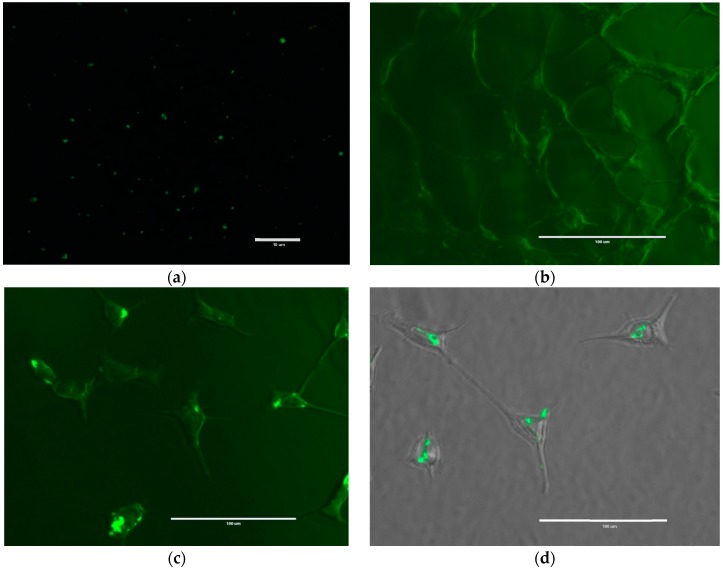
Fluorescein isothiocyanate (FITC)-labelled γ-PGA-chitosan NPs; (**a**) Labeled NPs suspended in deionised water at a concentration of 5 mg/mL. Magnification power ×1000. Scale bar, 10 µm; (**b**) HEK293 cells not exposed to labeled NPs used as a control; (**c**) HEK293 cells exposed to FITC-labeled NPs; (**d**) overlaying image of HEK293 cells exposed to FITC-labeled NPs. Magnification power ×400. Scale bar, 100 µm.

Fluorescent labeled NPs (fluorescein isothiocyanate (FITC)-γ-PGA-CH) were successfully formulated to investigate the cellular uptake of NPs. Particle size and Ad EE were not affected by the presence of the fluorescent dyes (result not shown). Figure 11 shows monodispersed FITC-labeled NPs as represented by well-spaced bright particles. These particles were suspended in deionized water at a concentration of 5 mg/mL, a drop was transformed to a clean glass slide and investigated under fluorescence microscopy. In addition, labeled NPs were further investigated to examine their behavior when introduced to HEK293 cells. This was performed in 6-well plates seeded with 3.0 × 10^5^ cells, left overnight to grow, and attached to the bottom of the plate at 37 °C with 5% CO_2_. The next day, the medium was removed and replaced with a medium containing FITC-labeled NPs at a concentration of 0.1 mg/mL.

After 24 h of incubation with NPs, the medium was removed and the cells were washed three times with PBS and fixed with ice-cold methanol for 10 min at −20 °C, and observed under microscope. The images from the inverted fluorescent microscope revealed that the labeled NPs were clearly attached/internalised on/into the cell, as non-attached labeled NPs were obviously washed out due to the washing cycles (Figure 11).

### 2.6. Cytotoxicity Assay

**Figure 12:**
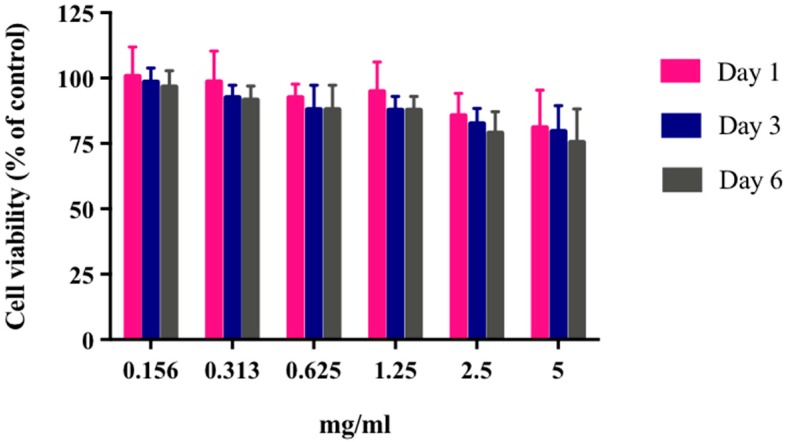
Effect of various concentrations of γ-PGA-CH NPs on HEK293A proliferation. Samples were incubated with cells for six days and cell proliferation was evaluated at day 1, day 3 and day 6 by MTT assay (*n* = 9).

The -(4,5-dimethylthiazol-2-yl)-2,5-diphenyltetrazolium bromide (MTT) assay is one of the most commonly used methods to assess the impact of NPs on cell viability. HEK293 cells were seeded in 96-well plates, and then incubated for 24 h at 37 °C in 5% CO_2_. Media were replaced with different concentrations of CH:γ-PGA NPs, i.e., 5, 2.5, 1.25, 0.625, 0.313, and 0.156 mg/mL. Cell proliferation was evaluated at day 1, 3, and 6, and compared to a control (cells not exposed to NPs). All nanoparticle concentration showed little to no cytotoxic effect on HEK293A cells, even after six days. When NPs were exposed to the cells, no significant difference was observed between them (*p* = 0.45). However, slow cell proliferation for the extreme concentration of NPs (5 mg/mL) was noticed, as the proliferation rate was about 75%. The lowest nanoparticle concentration showed no cytotoxicity throughout this experiment (Figure 12).

## 3. Discussion

The limitations associated with conventional anticancer drug formulations have led to numerous attempts in developing more effective therapies. In general, recombinant Ad vectors have many properties that make them useful for alternative cancer therapies [34]. However, the induction of the immune response to recombinant Ad has appeared as a significant concern in the success of anticancer therapies. Along with the encapsulation of biopolymer approaches, as used in this study, many other approaches are in place to reduce the activation of host-immune responses. These approaches include serotype switching, silencing mediators of hepatocyte injury, immunosuppression, use of high-dose vectors, and genetic manipulation of capsid-encoding sequences [35,36]. These approaches have many successful aspects, yet there are still limitations to their applicability. For instance, the immunosuppression approach has side effects that are unwanted in cancer-treatment clinical settings. Adenovirus immunostimulation is mediated mainly through viral capsid proteins, and is not dependent on transduction or viral gene expression [37]. To control Ad immunostimulatory properties, the encapsulation of Ad in γ-PGA NPs is an attractive approach.

The most widespread synthetic polymers for micro/nanoencapsulation are poly (lactic-co-glycolic acid) (PLGA), poly(glycolic acid) (PGA), and poly(lactic acid) (PLA). All these polymers have been approved by the U.S. Food and Drug Administration (FDA) for particular medical applications, as they are degradable [38]. Nevertheless, these synthetic polymers may reduce the surrounding pH during polymer degradation, which subsequently affects cellular function by generating a highly acidic microenvironment, thus limiting their in vivo application [39]. Increased attention has been turned to the use of biopolymers to overcome this problem. Naturally occurring biopolymers produced by living micro-organisms during cell growth may be the key, as they can be produced in the laboratory and they show obvious synthesizing flexibility. One of the most recently discovered biopolymers for nanoparticle applications is γ-PGA.

Biopolymer γ-PGA has been used in many therapeutic applications in the last few decades, as it is nonimmunogenic, biodegradable, and nontoxic. γ-PGA is an amorphous polymer with a T_g_ around 80 °C, as demonstrated by DSC and dynamic mechanical (DMTA) analyses (Figure 3 and Figure 4). It has a hydrophilic character (Figure 6), as expected on the basis of its chemical structure (Figure 1 and Figure 2), which justifies the presence of absorbed water in the sample. This is evidenced by the initial loss in the TGA curve (Figure 2) and by the elastic-modulus increase in DMTA measures (Figure 6), associated with an increase of sample rigidity due to absorbed-water evaporation. Additionally, the polymer has many beneficial properties, including a shielding surface that does not require chemical alteration or genetic manipulation, enhancement of vector stability, and eliminating nonspecific interaction. To reserve vector bioactivity, adenovirus encapsulation inside γ-PGA NPs can be performed in minor reaction conditions. Besides γ-PGA, CH and its derivatives are widely used for drug delivery; this has been attributed to their biodegradability and biocompatibility, as well as CH being able to be degraded into nontoxic products in vivo [40]. As the fabrication of γ-PGA NPs began in the last decade, there are very few data regarding their effect on mammalian cells. However, a study by Hsieh et al. (2005) demonstrated that the addition of γ-PGA and CH into the medium enhances hydrophilic properties and serum protein intake, and increases the attachment and growth of rat osteosarcoma cells [40]. Physical stability of the virus offered by γ-PGA against harsh environmental conditions was also demonstrated by our team in a previous study [41]. Some therapeutic agents have been encapsulated into polymeric NPs to protect them from neutralization in patients receiving repeated doses of this kind of treatment [22]. This application was the driving force for the development of an encapsulated Ad vector. After determining that Ad can be effectively encapsulated into γ-PGA-CH NPs, these NPs were characterized (Figure 7) and tested for their ability to avoid in vitro antibody neutralization. The Ad-loaded γ-PGA-CH NPs were fabricated using the ionic gelation technique. Encapsulation efficiency was 93% (Figure 8). The in vitro release behavior of Ad from γ-PGA-CH NPs was evaluated in physiological buffers at pH = 7 (Figure 9). Polymer degradation in an aqueous solution generates pores on the NP surface; thus, the release rate of smaller molecules is higher than that of bigger molecules. However, the entire released Ad retained full infectivity when introduced to HEK293 cells. The number of released Ad in this study was measured by an infectivity assay. In contrast, other mentioned studies evaluated the amount of released Ad by measuring the radioactivity of the radiolabeled Ad [42,43]. However, the observed Ad-release profiles in this study cannot be directly compared with others reported earlier, as different polymers, sizes, formulation recipes, and fabrication conditions were used for the preparation of Ad encapsulated in polymeric particles.

As an infectivity assay was used to measure the amount of released virus (Figure 8 and Figure 9), it is worth noting that all released Ad in our study retained full bioactivity, while the maximum bioactivity reported by Wang and coworkers (2007) was 11.4% [44]. A solvent evaporation process has been the most used method to date for encapsulating Ad. This procedure involves a series of critical damaging phases for Ad stability. Viral particles can be denatured and aggregated upon exposure to organic solvents. High shear forces may also cause irreversible viral inactivation through physical aggregation. Puapermpoonsiri et al. (2009) stated that organic solvent used in a solvent-evaporation method such as dichloromethane has a strong denaturing effect on the protein of the virus during the dichloromethane–water interface [45]. The results generated here are encouraging, as the formulation of encapsulated Ad vectors is feasible with retention of bioactivity due to the absence of organic solvent and less-damaging reaction conditions used in the ionic gelation method.

The present study also implies that a neutralizing antibody response against viral capsid proteins can be somewhat reduced by encapsulating Ad into γ-PGA NPs, as only 3.1% were detected by antiadenovirus antibodies (Figure 10). This finding is similar to that of the conjugation of Polyethylene Glycol (PEG) molecules to adenovirus capsids. Croyle et al. (2001) found that the modification of viral capsids with PEG can reduce the immune response generated against viral proteins [22]. However, a study by Shimizu and coworkers (2012) revealed that intravenous administration of PEG-coated Ad elicits anti-PEG antibodies, which reached the maximum level after five days of PEGylated Ad injection [46]. Moreover, unlike Ad encapsulation, modification of viral capsids with PEG can cause loss of infectivity due to the denaturation of viral surface proteins that mediate viral binding to the host cells [44]. However, these Ad-containing NPs have not been tested for generation of a cellular immune response. Thus, the exact nature of the response to these NPs must be extensively characterized in vitro and in vivo.

Further examination was conducted to evaluate the prospective kinetics of NPs towards the HEK293 cell line and cellular uptake. In addition, one clear advance allowing location and treatment of tumors more efficiently is through more precise targeting of tumor tissue with fluorescently labeled NPs. Radiation doses could be reduced through clearer imaging of the tumor. Drug wastage could similarly be reduced, along with concomitant side effects if the therapeutic agent was specifically directed at tumor tissue, with consequent minimization of collateral damage to adjacent healthy tissue. Conjugation of FITC to chitosan would give stability to the fluorescent dye that is usually applied for cellular uptake studies in vitro and in vivo. Inaccurate results may be obtained if FITC molecules are encapsulated inside the particle, as it is dissociated and subsequently released from the particle during the experiment. For that it is a very important consideration to certify that FITC-NPs measurement for NP kinetics and cellular uptake studies belong to the particle itself and not to the dissociated FITC molecules [47]. However, fluorescently labeled γ-PGA-CH NPs were successfully created in this study and introduced to mammalian cells. The images in Figure 11 from an inverted fluorescent microscope revealed that the labeled NPs were clearly attached/internalised on/into the cells. A study conducted by Kuo and coworkers (2011) suggested that γ-PGA could increase permeability by boosting the cellular uptake, possibly through its own specific receptor-mediated pathway [48].

There is no possibility that the produced NPs can be toxic to the mammalian cells used in this work. The obtained results showed that HEK293 cells continued to grow despite the presence of these particles even with extreme NP concentration (5 mg/mL) (Figure 12). The results were not surprising, as γ-PGA is known to be a nontoxic material. Besides γ-PGA, CH and its derivatives are widely used for drug delivery; this has been attributed to their biodegradability and biocompatibility. CH can also be degraded into nontoxic products in vivo [40]. As the fabrication of γ-PGA NPs started only in the last decade, there are very few data regarding their effect on mammalian cells. However, a study by Hsieh et al. (2005) demonstrated that the addition of γ-PGA and CH into the medium enhances NP hydrophilic properties. It can also increase serum protein intake as well as the attachment and growth of rat osteosarcoma cells [40].

## 4. Materials and Methods

### 4.1. γ-PGA Production, Purification, and Characterization

Bacterial γ-PGA was produced by *B. licheniformis* ATCC 9455a in a 5 L fermenter (Electrolab, Tewkesbury, UK) as reported earlier [49]. Infrared spectra were recorded on a Nicolet 380 FT-IR (Thermo Fisher Scientific Inc., Wilmington, DE, USA) with 32 scans and 4 cm^−1^ resolution. Samples were ground with KBr (Sigma Aldrich, Milano, Italy) (1 mg sample/100 mg KBr), and pelletized under pressure.

TGA measurements were carried out using a TA-TGA2950 (TA Instruments, New Castle, DE, USA). Analyses were performed at 10 °C/min from room temperature to 600 °C both under air and under nitrogen flow. DSC measurements were performed using a TA-DSC-Q100 (TA Instruments, New Castle, DE, USA) apparatus equipped with a liquid nitrogen cooling system (LNCS) accessory. Heating scans were run at 20 °C/min, from −100 °C to 200 °C in a helium atmosphere. Between heating scans, quench cooling was applied. Melting temperature (T_m_) was taken at the peak maximum of endotherm, while the glass-transition temperature (T_g_) was taken as the midpoint of the stepwise increase of the specific heat associated with the transition.

Wide-angle X-ray diffraction measurements (WAXS) were carried out at room temperature with a PANalytical X’Pert PRO diffractometer equipped with an X’Celerator detector for ultrafast data collection (PANalytical B.V., Almelo, The Netherlands). A Cu anode was used as X-ray source (KR radiation: λ = 0.154 18 nm, 40 kV, 40 mA), and ¼ divergence slit was used to collect the data in 2θ range from 2° to 60°. The degree of crystallinity (χ_c_) was evaluated as the ratio of the crystalline peak areas to the total area under the scattering curve.

Dynamic mechanical measurements (DMTA) were carried out in dual cantilever mode, at 3 °C/min and 3 Hz from −120 °C to 280 °C using a DMTA MkII (Polymer Laboratories Ltd., Church Stretton, UK). A strip (8 mm wide) cut from a glass fiber nonwoven mat (Whatman GF/C, Sigma Aldrich, Milano, Italy) was used to support the polymer. Polymer deposition was carried out by means of multiple immersions of the glass-fiber support in a 1% aqueous solution of Sample 2. A solvent-evaporation step (in oven under vacuum at 80 °C) was performed between consecutive immersions. The final glass-fiber strip coated with the polymer was subjected to DMTA measurement.

Rheological measurements were carried out at 25 °C using an Anton Paar MCR102 Rheometer (Anton Paar GmbH, Graz, Austria) with cone-plate CP50-1-SN19392 geometry (d = 0.102 mm, acquisition time 100 s, shear rate from 0.01 s^−1^ to 200 s^−1^).

Static water-contact angle measurements were performed on samples coated on glass slides (from a 0.8% aqueous solution) by means of a Laurell (WS-650-23NPP) spin coater (Laurell Technologies Corporation, North Wales, PA, USA) under ambient conditions. The contact-angle experiments were run with an optical contact angle and surface-tension meter KSV’s CAM 100 (KSV, Espoo, Finland) using distilled water. The water-drop profile images were collected every second in a time range of 0–30 s. Four drops per sample were analyzed.

The enantiomeric composition of the produced γ-PGA was investigated using HPLC (Thermo Scientific, Schwerte, Germany). The hydrolysis of γ-PGA was performed according to many studies [50,51]. Initially, the lyophilized polymer was resuspended in 6 M HCl (Sigma, Irvine, UK) at the concentration of 1 mg/mL, and hydrolyzed at 110 °C for 24 h. The hydrolyzed samples were then lyophilized again and dissolved in water and methanol (30:70) to the final concentration of 300 μg/mL. After that, 10 μL of the samples was injected into a Chirobiotic T column, 25 cm × 4.6 mm I.D., 5 μm particles (Astec, Sigma-Aldrich, Irvine, UK) at 25 °C using a mobile phase: water; methanol (Thermo Fisher Scientific, Altrincham, UK); and formic acid (Thermo Fisher Scientific, Altrincham, UK) (30:70:0.02, respectively) at a flow rate of 1 mL/min according to the manufacturer’s instructions. Chromatography was performed using a Dionex Ultimate 3000 series HPLC (Thermo Scientific, Schwerte, Germany) connected to an ultraviolet (UV) spectrometer (Thermo Scientific, Schwerte, Germany). The analytical software used was Chromeleon 7 (Thermo Scientific, Schwerte, Germany). In order to determine retention time, standards of d- and l-form of glutamic acid were first tested. After that, the mass of glutamic acid was used to determine the amounts of d- and l-glutamic acid by peak integration. Number average molar mass M*_n_* was determined by conventional aqueous-based GPC at Smithers Rapra in Shrewsbury, United Kingdom. An MZ Hema guard plus 2× Hema Linear column (Cognis Performance Chemicals Ltd., Southampton, UK) was used for analysis. The GPC experiments were carried out in 0.2 M NaNO_3_, 0.01 M NaH_2_PO_4_, at pH = 7, with a flow rate of 1 mL/min at 30 °C. The GPC system used was calibrated with polyacrylate standards. The data were collected and analyzed using Polymer Laboratories’ “Cirrus 3.0” software Supplied by Polymer Laboratories (Salop, UK).

### 4.2. Adenovirus Preparation

The HEK293 cell line was used to amplify Adenovirus-GFP (purchased from Applied Biological Materials, Inc., Richmond, BC, Canada). The adenovirus was purified using an Adenovirus mini purification viraKit (Virapur LLC, San Diego, CA, USA). Then, the adenovirus was titered using an Adeno-X Rapid Titer Kit (Clontech Laboratories, Inc., Mountain View, CA, USA). Initially, 0.5 mL of healthy, log-phase HEK 293 cells (2.5 × 10^5^ cells/mL) suspended in standard growth medium (DMEM + 10% FBS + antibiotics) was seeded in each well of a 24-well plate. Using PBS as diluent, 10-fold serial dilutions of the viral sample were prepared from 10^−2^ to 10^−7^ mL. After that, 50 µL of viral dilution was added to each well. Subsequent to incubation for 48 h at 37 °C with 5% CO_2_, cell medium was aspirated. Then, cells were fixed by adding 0.5 mL ice-cold 100% methanol to each well, and the plate was incubated at −20 °C for 10 min. Subsequently, methanol was aspirated and the wells were rinsed 3 times with PBS + 1% BSA. The final rinse was aspirated from the wells and 0.25 mL of (1:1000) diluted mouse antihexon antibody was added to each well, then incubated for 1 h at 37 °C. The antihexon antibody was aspirated, and cells were washed with 1 mL PBS + 1% BSA. Then, 0.25 mL of (1:500) diluted rat antimouse antibody (conjugate) was added to each well, and incubated for 1 h at 37 °C. Prior to removing rat antimouse antibody, a diaminobenzidine (DAB) working solution was prepared by diluting 10X DAB substrate 1:10 with 1X stable peroxidase buffer. The rat antimouse antibody dilution was subsequently aspirated and again rinsed with 1 mL PBS + 1% BSA. After removing the final PBS + 1% BSA rinse, 500 µL of DAB working solution was added to each well, and incubated at room temperature for 10 min. Finally, the DAB solution was aspirated and 1 mL PBS was added to each well. The mean of a minimum of 3 fields of brown positive cells were counted using a microscope with a 20× objective. Calculations of infectious units (ifu)/mL for each well were performed using the manufacturer’s manual.

### 4.3. Adenovirus Encapsulation

Chitosan (50–190 kDa) with 75–85% deacetylation (purchased from Sigma, UK), was dissolved in a 0.1 M glacial acetic acid (Acros, UK) solution (pH = 5.5) to a final concentration of 10 mg/mL. The prepared solution was left to stir overnight, followed by a purification process in a dialysis tube (Specturm, Irving, TX, USA) with a 10 kDa cut-off. After 3 days of dialysing, CH was precipitated by increasing the pH to 10. The precipitant was washed 3 times and freeze-dried.

Solutions of CH and γ-PGA were prepared before forming NPs. Purified CH was dissolved in 0.1 M glacial acetic acid at concentration of 10 mg/mL and left to stir overnight on a magnetic stirrer. The following day, the CH solution was autoclaved under pressure of 10 psi for 20 min at 121 °C. Subsequently, the solution was passed through a filter (0.45 μm) to eliminate nondissolved particles. Finally, 0.05% of CH solution was obtained with pH = 5.5. To prepare the γ-PGA solution, 10 mg/mL of γ-PGA in deionized water was thermally depolymerized at 100 °C for 180 min. Then, 0.15% of γ-PGA in 0.1% sodium tripolyphosphate (TPP) solution was prepared at pH = 6.8. Multi-ion-crosslinked Ad-loaded NPs were fabricated by a simple ionic-gelation method as described previously [52], with some modifications. In brief, 3.0 × 10^8^ PFU/mL of Ad in 200 μL PBS solution was added to 7.8 mL of CH and left to stir on ice for 30 min. Subsequently, 2 mL of γ-PGA was added drop-wisely using a digital pipette (Thermo Fisher Scientific, Waltham, MA, USA) with a flow rate of 2 mL/min. The solution was left to mix on a magnetic stirrer. Suspension was spontaneously formed after 1 h of crosslinking. The obtained particles were transferred to a sterile centrifuged tube containing a bed of glycerol (Sigma, UK), and kept in the fridge to set for 30 min. After that the particles were collected by centrifugation at 8000× *g* for 80 min at 4 °C. To remove the Ad that was adsorbed to the surface of the NPs or free in the supernatant, the obtained particles were collected by a washing and centrifugation (Sigma, UK) step at 8000× *g* for 80 min at 4 °C. The pellet was then resuspended in 2.5% of trehalose (Sigma, UK), which can be used directly or freeze-dried. Another sample was prepared under the same circumstances without γ-PGA as a control. To determine the EE of Ad in γ-PGA-CH NPs, the amount of free Ad in supernatants was assayed by the Adeno-X Rapid Titer Kit as shown below:

(1) $$\mathrm{EE}\left(\%\right)=\frac{\mathrm{Ad}\;\mathrm{in}\;\mathrm{control}-\mathrm{free}\;\mathrm{Ad}\;\mathrm{in}\;\mathrm{supernatant}}{\mathrm{Ad}\;\mathrm{in}\;\mathrm{control}}\times 100$$

SEM (Zeiss EVO50, Carl Zeiss Ltd., Cambridge, UK) analysis was performed to determine the morphological surface structure of freeze-dried NPs. Samples were prepared by freeze-drying. The obtained fine dry powder samples were placed on an aluminum stub with a double-sided carbon tape, then coated with gold. Analyses were conducted using an SEM and microphotographs were analyzed using SmartSEM software V05.06 (Carl Zeiss Ltd., Cambridge, UK). The size and zeta potential of γ-PGA nanoparticles synthesized during this project were continuously measured using dynamic light scattering from Malvern (HeNe laser, Malvern, UK).

### 4.4. In Vitro Virus Release from Chitosan-γ-PGA NPs

The Ad release profile from produced NPs (0.05:0.15 CH:γ-PGA ratio) was obtained in a DMEM medium (pH = 7.0), supplemented with 10% fetal bovine serum (FBS), 1% l-glutamine, and 1% antibiotic antimycotic solution (Sigma, UK). The pellet after the centrifugation and washing step was resuspended in the medium and incubated in an orbital shaker at 37 °C under agitation (150 rpm). At predetermined time intervals, NP suspension was centrifuged 8000× *g* for 80 min, and supernatant was removed and assayed by plaque-forming assay to determine the amount of the released viruses. Then, the NP pellet was again resuspended in the fresh medium and agitated; the amount of viruses released from the produced NPs was expressed as a percentage with respect to the total amount of viruses encapsulated into the NPs. Release results were obtained daily in triplicate for a time course of 10 days.

### 4.5. Fluorescent Labeled NPs

To investigate NP cellular uptake, it was vital to label the particle with fluorescent dye. To label NPs with FITC, a method described in a previous study [53] was followed. In brief, 20 mg of FITC (Sigma, UK) in 20 mL dehydrated methanol was added to 20 mL 1% *w*/*v* CH in 0.1 M acetic acid solution. Then, it was left for 3 h to react in the dark at room temperature. The FITC-labeled chitosan was precipitated by raising the pH to 10 with 1 M NaOH. The unreacted FITC was washed away with deionized water and separated by centrifugation until no fluorescence was detected in the supernatant. The FITC-CH was dissolved in acetic acid and dialyzed in 5 L of deionized water for 3 days under darkness, with water being replaced every day. The nanoparticles were then fabricated using the same method as described above.

### 4.6. Adenovirus Immunogenicity 

An indirect ELISA test was carried out to check whether NPs have the potential to shield Ad from neutralizing antibodies or not. In brief, flat-bottomed 96-well plates were coated for 1 h at 37 °C with 50 µL of (1:1000) diluted antihexon antibody (primary antibody). Following 3 washes with PBS, plates were blocked with 100 μL of PBS + 2% BSA at 37 °C for 1 h. Plates were then washed 3 times with PBS. Naked Ad for a standard curve was prepared by a 10-fold serial dilution using PBS from 9.8 × 10^6^, to 9.8 × 10^2^ ifu/mL, and each dilution was added at 50 µL per well. The same amount of encapsulated Ad was also diluted down and applied to coat the 96-well plate, as above. After 1 h at room temperature, viruses were washed 3 times, followed by the addition of 50 µL more of (1:1000) diluted antihexon antibody (primary antibody) and incubated for 1 h at 37 °C. Subsequently, wells were rinsed 3 times with PBS, and 50 µL of (1:500) diluted rat antimouse antibody (conjugate antibody) was added. Plates were incubated for 1 h at room temperature and protected from direct light. After washing 3 times with PBS, 50 µL per well of tetramethylbenzidine (TMB) (Sigma, UK) substrate was added. After 4 min of incubation in the dark, the reaction was stopped upon the addition of 2 M HCl. The absorbance of the resulting solution was measured at 450 nm in a Multiskan Ascent (Thermo Labsystems, Altrincham, UK) plate spectrophotometer.

### 4.7. Cytotoxicity Assay

The stock MTT solution was prepared by dissolving 3-(4,5-dimethylthiazol-2-yl)-2,5-diphenyltetrazolium bromide (Sigma, Gillingham, UK) in PBS to a final concentration of 5 mg/mL. Cells were cultured in 96-well plates (5.0 × 10^3^/well) and were left for 24 h to be attached to the bottom of the incubator at 37 °C with 5% CO_2_. Cells were exposed to different concentrations of NPs prepared by a 2-fold serial dilution using a fresh culture medium, i.e., 5, 2.5, 1.25, 0.625, 0.313, and 0.156 mg/mL. There was also a positive control of cells that was not exposed to NPs. After 24 h, the medium in each well was replaced with 200 μL of fresh medium containing different concentrations of NPs and incubated at 37 °C with 5% CO_2_. NP cytotoxicity was investigated at day 1, 3, and 6 by adding 50 μL of MTT (5 mg/mL) to the wells; the plates were protected from direct light. The solution was removed after incubation for 4 h at 37 °C. Subsequently, 80 μL of 99.9% DMSO and 20 μL glycine buffer (0.1 M glycine and 0.1 M NaCl equilibrated to pH = 10.5) (Sigma) were added. The absorbance of the resulting solution was measured at 540 nm in a Multiskan Ascent (ThermoLabsystems, Altrincham, UK) plate spectrophotometer.

### 4.8. Statistical Analysis

All results were statistically analyzed using the Student’s *t*-test and a one-way ANOVA in statistical package GraphPad Prism version 6.03 (GraphPad Software, Inc., La Jolla, CA, USA). *p* ≤ 0.05 was considered to be statistically significant.

## 5. Conclusions

The novel potential of γ-PGA NPs as a virus carrier was evaluated in an ionic complexation. The virus was encapsulated into the polymeric NPs through ionic interactions, thus avoiding the need for surface modification, organic solvent, or covalent linkage between the virus and the polymer. The virus-encapsulated γ-PGA ionic-complex NPs may also act as a new vector for Ad and facilitate its controlled release or internalization in malignant tissue and cells. Up to 92% of the viruses were encapsulated into the nanoparticles. The particle size of virus-loaded nanoparticles was about 485 nm, and the nanoparticles had net-negative zeta potential. Upon suspending the virus-loaded nanoparticles in the release medium, the virus was leached from the nanoparticles showing sustained release, and the viruses released from the nanoparticles were bioactive and fully infective. The immunogenicity study revealed that only 3.1% of the encapsulated viruses were detected by neutralizing antibodies. Using nanoparticles as a drug-delivery system based on γ-PGA, as detailed in this work, leads to the prediction that they could be crucial in the future due to their unique properties and safety features. More systematic studies in vivo must be performed to establish the performance and consistency of these nanoparticles in the blood or lymphatic fluids.
